# Baseline predictors of progression of Parkinson’s disease in a sample of Egyptian patients: clinical and biochemical

**DOI:** 10.1186/s41983-022-00445-1

**Published:** 2022-01-15

**Authors:** Asmaa Helmy, Eman Hamid, Mohamed Salama, Ahmed Gaber, Mahmoud El-Belkimy, Ali Shalash

**Affiliations:** 1 Department of Neurology, Faculty of Medicine, Ain Shams University, 168 Elnozha St, Saint Fatima Square, Heliopolis, Cairo, Egypt; 2 Institute of Global Health and Human Ecology (I-GHHE), The American University in Cairo, Cairo, Egypt; 3 Faculty of Medicine, Al-Mansoura University, Mansoura, Egypt

**Keywords:** Parkinson’s disease, Predictors, Progression, Egyptian, COVID-19

## Abstract

**Background:**

Clinical progression of Parkinson’s disease (PD) is highly heterogeneous, and its predictors are generally lacking. Identifying predictors of early disease progression is important for patients’ management and follow-up. The current study aims to identify clinical, neuroimaging and biochemical baseline predictors of motor progression in patients with PD. Forty-five PD patients were assessed at baseline, 6 months and 1 year using MDS-UPDRS total and subscores, Hoehn and Yahr (H&Y), Schwab and England (S&E), International Physical Activity Questionnaire (IPAQ). Baseline New Freezing of Gait Questionnaire (NFOG-Q), Berg Balance Scale (BBS), Ten-Meter Walking Test (10-MWT)**,** and Time Up and Go Test (TUG), Non-Motor Symptoms Scale (NMSS), Beck Depression Inventory (BDI), PD questionnaire 39 (PDQ-39), MRI brain, uric acid, lipid profile and glycated hemoglobin were performed.

**Results:**

Significant worsening of MDS-UPDRS total, part III scores, H&Y, S&E and IPAQ (*p* < 0.001) was detected. One-year progression of H&Y and S&E were significantly correlated to disease duration (*p* = 0.014, *p* = 0.025, respectively). Progression of H&Y was correlated to baseline TUG (*p* = 0.035). S&E progression was correlated to baseline MDS-UPDRS total score (rho = 0.478, *p* = 0.001) and part III (rho = 0.350, *p* = 0.020), H&Y (rho = 0.401, *p* = 0.007), PIGD (rho = 0.591, *p* < 0.001), NFOG-Q (rho = 0.498, *p* = 0.001), and TUG (rho = 0.565, *p* = 0.001). Using linear regression, there was no predictors of clinical progression among the used baseline variables.

**Conclusion:**

Despite the significant motor and physical activity progression over 1 year that was correlated to baseline motor and gait severity, but without predictive value, further similar and longitudinal studies are warranted to detect predictors of early progression and confirm findings.

**Supplementary Information:**

The online version contains supplementary material available at 10.1186/s41983-022-00445-1.

## Background

Parkinson’s disease (PD) is the second most common progressive neurodegenerative disorder, affecting 1% of people older than 60 years, with incidence rates of 8–18 per 100,000 person-years in prospective population-based studies [1]. PD is a complex disease with clinical, genetic, and molecular grounds. Clinical progression is therefore highly heterogeneous across individuals, and predictors of individual progression are generally lacking [2, 3].

Most studies on the annual rate of changes of motor symptoms in early PD show large inter-individual variation, even in the first year of observation, pointing towards different progression trajectories [4]. Therefore, indicators for disease progression are warranted. Currently, as reported in smaller studies with a small number of parameters, the most important predictors for worse motor progression are age and motor disability at baseline [5]. Recent findings suggest that cardiovascular risk factors contribute to a more severe PD phenotype, but a multimodal approach is lacking [6]. The difficulty in identifying early diagnostic criteria for PD depends on the fact that no real biomarker can yet predict illness onset [7]. Therefore, investigating the available laboratory tests and MRI brain basic findings is more feasible and require further research.

In this present study, we assessed the predictors of motor progression of PD over 1 year either clinical (baseline characteristics), neuroimaging or biochemical (serum uric acid, lipid profile, glycated hemoglobin (HbA1c)).

## Methods

In this prospective cohort study, a convenient sample of 75 recruited patients diagnosed with PD according to International Parkinson and Movement Disorders Society (MDS) diagnostic criteria [8], out of them, 45 patients completed the assessments at baseline, 6 months and after 1 year. Patients were recruited from Movement Disorder Outpatient Clinic at Ain Shams University hospitals in the period between July 2019 and December 2020. Patients with atypical or acquired Parkinsonism or underwent functional brain surgery were excluded.

An informed consent for participation in the study was obtained from all participants following a full explanation of the protocol, that was approved by ethical committee of faculty of medicine, Ain Shams University (FWA 000017585 in 10th of July 2019), according to the Declaration of Helsinki. This study was registered in clinicalTrial.gov, NCT04062279.

Comprehensive medical history, neurological examination, laboratory testing, as well as brain imaging were done initially, followed by assessment scales at baseline, 6 months, and at 1 year. They included motor assessment using MDS-UPDRS during OFF state, Hoehn and Yahr, Schwab and England Activities of Daily Living Scale [9], baseline gait assessment during OFF state by New Freezing of Gait Questionnaire (NFOG-Q) [10], Berg Balance Scale (BBS) [11], 10-Meter Walking Test (10-MWT) [12] and Time Up and Go Test (TUG) [13]; physical activity through International Physical Activity Questionnaire short form (IPAQ) [14], baseline assessment of non-motor symptoms using the Non-Motor Symptoms Scale (NMSS) [15]; depression assessment by the Arabic version of Beck Depression Inventory (BDI) [16], as well as quality of life testing using the Arabic version of PD questionnaire 39 (PDQ-39) [17]; and cognitive assessment at baseline was done by Mini-Mental State Examination (MMSE) [18]. Levodopa equivalent daily dosage (LEDD) was calculated at baseline as the sum of the daily dose of all dopaminergic agents [19]. Mean difference was calculated as the difference between baseline and follow-up scores. Percentage change was equal to the change in value divided by the absolute value of the original value, multiplied by 100.

Serum level of uric acid, lipid profile and HbA1c were measured initially at baseline. Magnetic resonance imaging (MRI) brain to assess white matter hyperintensities lesions (WMHL) was done using Fazekas scale scoring from Fazekas 0: no or a single punctate WMHL, to Fazekas 3: large confluent lesions [20], Scheltens score [21] for evaluation of WMHL number, size and localization (periventricular, deep white matter, basal ganglion, infratentorial) scoring from 0 = no abnormalities, to 6 = confluent lesions [22]. In addition to the whole-group data analysis, comparisons between patients subtypes were performed according to motoric subtypes (TD vs non-TD) [23], male vs female gender, early-onset vs late-onset PD, and mild vs moderate and advanced stages [24].

### Statistical analysis

Data analysis was done by IBM SPSS software package version 25.0 (Armonk, NY: IBM Corp). The normality test was done using Kolmogorov–Smirnov test. Qualitative data were described in frequency and percentage. Quantitative data were described in mean ± standard deviation. Repeated measures ANOVA (RMANOVA) was used for normally distributed continuous variables of the three-point assessments, while Friedman test was used with post hoc analysis by Wilcoxon tests and Bonferroni correction (*p* value is significant if < 0.017) for ordinal variables or not normally distributed continuous ones. Pearson correlation coefficient was used to correlate continuous normally distributed variables, while Spearman correlation was used in case of continuous not normally distributed variables. Mann–Whitney test was used in subgroup analysis. The significance was judged at confidence interval (CI) set to 95% and statistical significance at *P* < 0.05 (except for correlation coefficient in which Bonferroni adjustment was done and calculated according to the following formula α/*m* where α is the desired overall alpha level and *m* is the number of hypotheses) [25]. Linear regression was used to investigate the predictors of clinical progression over 1 year. Sample size was calculated by standard formula to give the confidence interval of 95% and ± 5% margin of error.

## Results

Forty-five patients (34 males, 75.6%) completed the follow-up assessments up to 1-year. Mean age was 56.46 ± 9.32 (ranging from 32 to 78 years old), age of onset was 51.32 (± 9.67) years (ranging 26–74 years), and mean duration was 4.9 (± 3.09) years (from 0.25 to 14). Mean disease stage Hoehn and Yahr was 2.57 ± 0.71 (ranging from 1.5 to 4). Twenty-two patients were either illiterate (14 patients) or just read and write (8 patients). At baseline, 29 patients (64.4%) were depressed using BDI ((14 (31.1%) mild, 8 (17.8%) moderate and 7 (15.6%) severe depression). At baseline, 34 (75.5%) patients were tremor dominant (TD) while 9 (20%) patients were akinetic rigid and only 2 (4.44%) were indeterminate. Fifteen (33.33%) patients were young-onset PD (YOPD) (AOO < 50 years) and 30 (66.67%) patients were late-onset PD (LOPD) (AOO > 50 years). Table 1 and Additional file 1: Table S1 show detailed baseline characteristics of enrolled PD patients including clinical, laboratory and brain imaging. The 1st follow-up (at 6 months) of 35 patients (77.8%) and 2nd follow-up (at 1 year) of 44 patients (97.8%) were during the COVID-19 pandemic.

**Table 1:** Baseline demographic data and clinical characteristics of PD patients

	Mean (SD)/frequency (%)	Range
Age (years)	56.45 (9.31)	32–78
Gender		
Male	34 (75.6%)	
Female	11 (24.4%)	
AOO (years)	51.32 (9.67)	26–74
DOI (years)	4.90 (3.1)	0.25–14
MDS-UPDRS Total score OFF	81.75 (33.02)	29–176
MDS-UPDRS-I	15.25 (7.29)	1–35
MDS-UPDRS-II	18.48 (10.19)	0–44
MDS-UPDRS-III OFF	47.89 (19.82)	13–101
Hoehn and Yahr OFF	2.53 (0.69)	1.5–4
Schwab and England ADL OFF	76.14 (13.16)	30–90
TUG OFF	19.21 (16.31)	7.86–82.80
(10-MWT) Comfortable speed OFF (m/s)	0.77 (0.33)	0.10–1.29
(10-MWT) Maximum speed OFF (m/s)	1.031 (0.43)	0.12–1.75
BBS OFF	45.77 (9.91)	14–56
NFOG-Q OFF	10.57 (10.10)	0–29
IPAQ	2058.77 (821.20)	636–3942
MMSE	26.45 (3.17)	16–30
NMSS total score	61.57 (44.19)	4–205
BDI	17.61 (9.87)	0–51
PDQ-39	37.08 (18.68)	2.6–76.7

*AOO*  age of onset, *DOI*  duration of illness, *MDS-UPDRS*  Movement Disorder Society—Unified Parkinson’s Disease Rating Scale, *ADL*  activities of daily living, *TUG* Time Up and Go Test, *10-MWT*  10-Meter Walking Test, *BBS*  Berg Balance Scale, *NFOG-Q*  New Freezing of Gait Questionnaire, *IPAQ*  International Physical Activity Questionnaire, *MMSE*  Mini-Mental State Examination, *NMSS*  Non-Motor Symptoms Scale, *BDI*  Beck Depression Inventory, *PDQ_39*  Parkinson’s Disease Questionnaire-39

**Fig. 1:**
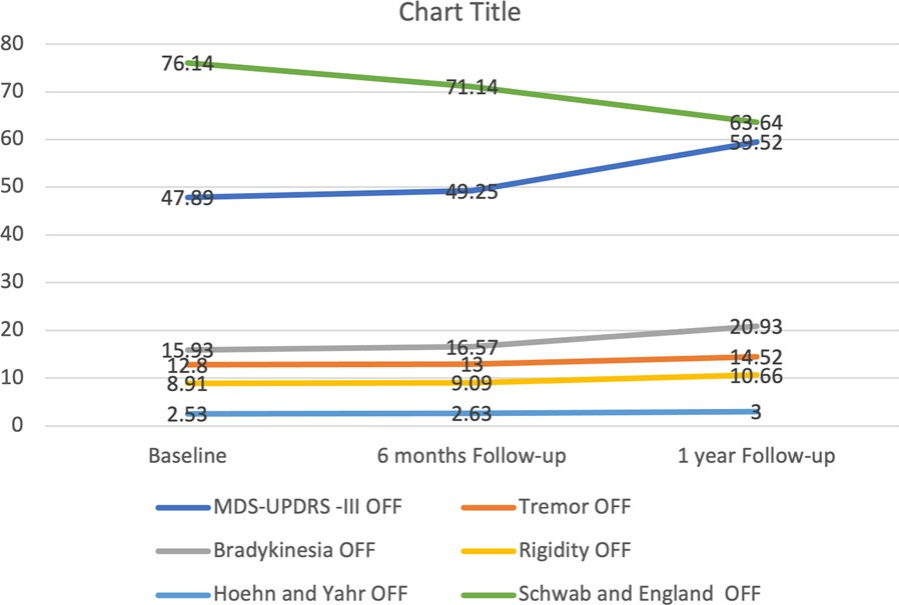
Motor progression of PD patients over 1-year follow-up

There were significant worsening in MDS-UPDRS total, part III scores, Hoehn and Yahr and Schwab and England ADL scores (*p* < 0.001) at 1-year follow-up. Also, there was a significant worsening of axial, rigidity (*p* < 0.001), tremor (*p* < 0.001) scores at 1-year assessment. Also, IPAQ showed highly significant worsening at 1-year follow-up (*p* < 0.001) (Table 2, Fig. 1).

**Table 2:** Progression of motor and physical activity over 6-month and 1-year follow-up

	Baseline	6-month FU	1-year FU	RMANOVA	Baseline versus 6 months	6 months versus 1 year	Baseline versus 1 year
	Mean (SD)	Mean (SD)	Mean (SD)	*p*			
MDS-UPDRS total score OFF	81.75 (33.02)	86.50 (29.66)	105.86 (33.21)	< 0.001*	0.009*	< 0.001*	< 0.001*
MDS-UPDRS-I	15.25 (7.29)	16.25 (6.24)	21.34 (6.01)	< 0.001*	0.149	< 0.001*	< 0.001*
MDS-UPDRS-II	18.48 (10.19)	20.70 (8.62)	25.02 (9.14)	< 0.001*	0.013*	< 0.001*	< 0.001*
MDS-UPDRS-III OFF	47.89 (19.82)	49.25 (18.95)	59.52 (21.65)	< 0.001*	0.288	< 0.001*	< 0.001*
Tremor OFF	12.80 (7.47)	13.00 (7.56)	14.52 (8.03)	< 0.001*	1	0.001*	0.002*
Bradykinesia OFF	15.93 (7.57)	16.57 (7.08)	20.93 (7.68)	< 0.001*	0.356	< 0.001*	< 0.001*
Rigidity OFF	8.91 (4.15)	9.09 (3.68)	10.66 (3.95)	< 0.001*	1	< 0.001*	< 0.001*
Axial OFF^a^	13.93(6.88)	14.70 (6.13)	18.23 (6.86)	< 0.001*	**0.014**	< 0.001*	< 0.001*
PIGD OFF^a^	8.55 (5.72)	8.84 (5.26)	10.70 (5.54)	< 0.001*	0.193	< 0.001*	< 0.001*
Motor complication total score^a^	4.93 (4.48)	5.89 (4.18)	6.91 (4.23)	< 0.001*	**0.004**	< 0.001*	< 0.001*
Hoehn and Yahr OFF^a^	2.53 (0.69)	2.63 (0.75)	3.00 (0.87)	< 0.001*	0.024	< 0.001*	< 0.001*
Schwab and England ADL OFF^a^	76.14 (13.16)	71.14 (16.46)	63.64 (16.72)	< 0.001*	** < 0.001**	< 0.001*	< 0.001*
IPAQ	2058.77 (821.20)	1622.21 (779.69)	1186.73 (605.78)	< 0.001*	< 0.001*	< 0.001*	< 0.001*

Bold values are significant

*MDS-UPDRS*  Movement Disorder Society—Unified Parkinson’s Disease Rating Scale, *PIGD*  postural instability and gait disorder, *ADL*  activities of daily living, *IPAQ*  International Physical Activity Questionnaire, *RMANOVA*  repeated measures ANOVA

^a^Friedman’s test was used and post hoc analysis was done by Wilcoxon tests with Bonferroni correction (*p* value is significant if < 0.017)

^*^p value is significant if < 0.05

According to Hoehn and Yahr staging, 34 (75.6%) patients had early (mild) PD (stages 1–2.5) at baseline vs 18 (40.9%) patients at 1-year follow-up, while 5 (11.1%) had advanced PD (stages 4–5) at baseline vs 14 (31.8%) at 1-year follow-up (*p* 0.004).

It was noticed that the motor progression was more obvious in the second half of follow-up year especially in MDS-UPDRS part I, III, and MDS-UPDRS subscores, as well as Hoehn and Yahr staging (Table 2 and Fig. 1). The mean rate of motor progression at 1 year was 26.93% for MDS-UPDRS III, 18.78% for Hoehn and Yahr and 17.19% of Schwab and England ADL in the Off state (Additional file 1: Table S2).

Comparing 34 patients with early/mild PD (Hoehn and Yahr ≤ 2.5) vs 11 patients with moderate and advanced PD stages (Hoehn and Yahr 3, 4,5), baseline scores of MDS-UPDRS total, part II and part III were higher in advanced and moderate stages (*p* < 0.001). Moreover, NFOG-Q, TUG, IPAQ and Schwab and England ADL baseline scores were also higher in advanced and moderate stages (*p* < 0.001). On the other hand, disease progression was not significant in all parameters between early and advanced stages except for Schwab and England ADL (*p* = 0.01) (Additional file 1: Table S3).

Male and female patients were matched regarding age, disease duration and severity. Compared to males, female patients had significantly higher NFOG-Q (*p* = 0.03) and PIGD (*p* = 0.03), prolonged TUG (*p* = 0.03), slower 10-MWT comfortable (*p* = 0.02) and maximum speed (*p* = 0.03), and higher serum HDL level (*p* = 0.02). On the other hand, disease progression was not significant between males or females in all parameters (Additional file 1: Table S4).

Patients with non-TD PD subtype presented earlier in life compared to TD PD (*p* = 0.046) with longer DOI (*p* = 0.039). Expectedly, the MDS-UPDRS tremor subscore was significantly higher in TD PD (*p* < 0.001) while PIGD was higher in non-TD (*p* = 0.005). Patients with TD PD were significantly faster than the non-TD PD subtype (*p* = 0.039), with lower freezing of gait scores (*p* = 0.001). and better quality of life (*p* = 0.017) compared to non-TD PD. Regarding disease progression, only Hoehn and Yahr and IPAQ showed significantly worse progression among patients with the non-TD type (*p* = 0.040 and 0.012, respectively) (Additional file 1: Table S5).

Comparing EOPD (15 patients) with LOPD (30 patients), the former had significantly shorter disease duration (*p* = 0.040), higher HbA1C (*p* = 0.01) and higher Scheltens total scores (*p* = 0.04), respectively. Yet, there was no significant difference regarding clinical progression between the two groups (Additional file 1: Table S6).

One-year progression of Hoehn and Yahr and Schwab and England ADL were significantly correlated to disease duration (rho = 0.367, *p* = 0.014; and rho = 0.337, *p* = 0.025, respectively). Progression of Hoehn and Yahr was correlated to baseline TUG (rho = 0.319, *p* = 0.035). Schwab and England ADL progression was correlated to baseline of MDS-UPDRS total score (rho = 0.478, *p* = 0.001), PIGD (rho = 0.591, *p* < 0.001), NFOG-Q baseline (rho = 0.498, *p* = 0.001)**,** TUG (rho = 0.565, *p* = 0.001), MDS-UPDRS part III (rho = 0.350, *p* = 0.020), and Hoehn and Yahr (rho = 401, *p* = 0.007) (Table 3).These correlations were non-significant after Bonferroni correction (*p* < 0.003) except for Schwab and England ADL progression correlation with baseline MDS-UPDRS total score, PIGD, TUG and NFOG-Q (Table 3, Additional file 1: Table S7).

**Table 3:** Correlations between progression of motor and physical activity with baseline demographic and clinical characteristics

Mean difference between baseline and 1-year follow-up		Age	AOO^a^	DOI^a^	Baseline MDS-UPDRS-OFF-Total score	Baseline MDS-UPDRS-III	Baseline PIGD OFF^a^	Baseline Hoehn and Yahr OFF^a^	Baseline TUG OFF^a^	Baseline NFOG-Q OFF^a^
Δ MDS-UPDRS total score OFF	Pearson	− 0.024	− 0.060	0.073	− 0.170	− 0.045	− 0.029	0.152	0.078	− 0.025
	Sig	0.876	0.698	0.640	0.269	0.773	0.851	0.324	0.614	0.874
Δ Axial OFF^a^	Spearman	0.059	0.024	− 0.015	− 0.168	− 0.143	− 0.172	0.011	0.070	− 0.016
	Sig	0.702	0.879	0.923	0.275	0.355	0.263	0.945	0.652	0.918
Δ PIGD OFF^a^	Spearman	− 0.033	− 0.053	0.010	− 0.110	− 0.044	− 0.226	0.078	0.117	− 0.047
	Sig	0.830	0.734	0.951	0.476	0.775	0.140	0.616	0.450	0.764
Δ Motor complication total score^a^	Spearman	− 0.186	− 0.281	0.084	− 0.194	− 0.095	− 0.183	− 0.193	− 0.273	− 0.137
	Sig	0.227	0.065	0.586	0.208	0.539	0.234	0.210	0.073	0.375
Δ Hoehn and Yahr OFF^a^	Spearman	0.006	− 0.102	**0.367**	0.204	0.157	0.284	0.181	**0.319**	0.252
	Sig	0.970	0.509	**0.014**	0.184	0.309	0.062	0.239	**0.035**	0.099
Δ Schwab and England ADL OFF^a^	Spearman	0.098	− 0.042	**0.337**	**0.478**	**0.350**	**0.591**	**0.401**	**0.565**	**0.498**
	Sig	0.526	0.785	**0.025**	**0.001**	**0.020**	** < 0.001**	**0.007**	** < 0.001**	**0.001**
Δ IPAQ	Pearson	0.055	0.199	− 0.222	− 0.073	− 0.045	− 0.110	0.110	− 0.042	− 0.231
	Sig	0.725	0.195	0.148	0.639	0.773	0.477	0.478	0.788	0.131

Bold values are significant

*AOO* age of onset, *DOI*  duration of illness, *LEDD*  levodopa equivalent daily dose, *MDS-UPDRS*  Movement Disorder Society—Unified Parkinson’s Disease Rating Scale, *PIGD* postural instability and gait disorder, *ADL* activities of daily living, *IPAQ*  International Physical Activity Questionnaire

^a^Spearman’s correlation was used

^*^p value is significant if < 0.05

^**^After Bonferroni correction, *p* value is significant if < 0.003

One-year progression of MDS-UPDRS total score, PIGD, and axial were inversely correlated to baseline NMSS total score (*r* = − 0.376, *p* = 0.012; rho = − 0.371, *p* = 0.013; rho = − 0.392, *p* = 0.009, respectively). Schwab and England ADL progression was directly correlated to baseline of PDQ-39, BDI and NMSS total score (rho = 0.426, *p* = 0.004; rho = 0.302, *p* = 0.046 and rho = 302, *p* = 0.046, respectively) and inversely correlated to baseline MMSE (rho = − 399, *p* = 0.007). Progression of motor complications and IPAQ were correlated to baseline of IPAQ (rho = 0.379, *p* = 0.011; rho = 675, *p* < 0.001, respectively). Progression of axial and motor complications were inversely correlated to baseline BDI (rho = − 298, *p* = 0.49; rho = − 360, *p* = 0.017, respectively). These correlations were non-significant after Bonferroni correction (*p* < 0.003) except physical activity worsening with baseline physical activity (*p* < 0.001) (Table 4).

**Table 4:** Correlations between progression of motor and physical activity with baseline cognitive, NMSS and quality of life

Mean difference between baseline and 1-year follow-up		Baseline MMSE^a^	Baseline NMSS total score	Baseline BDI	Baseline PDQ-39	Baseline IPAQ	Baseline LEDD
Δ MDS-UPDRS total score OFF	Pearson	0.046	− 0.376*	− 0.220	− 0.211	0.125	− 0.133
	Sig	0.765	**0.012**	0.152	0.170	0.420	0.391
Δ Axial OFF^a^	Spearman	− 0.039	**− 0.392**	**− 0.298**	− 0.298	0.231	− 0.125
	Sig	0.802	**0.009**	**0.049**	0.050	0.131	0.418
Δ PIGD OFF^a^	Spearman	− 0.102	**− 0.371**	− 0.289	− 0.222	0.152	− 0.115
	Sig	0.510	**0.013**	0.057	0.148	0.325	0.459
Δ Motor complication total score^a^	Spearman	0.002	− 0.225	**− 0.360**	− 0.116	**0.379**	0.006
	Sig	0.991	0.141	**0.017**	0.454	**0.011**	0.971
Δ Hoehn and Yahr OFF^a^	Spearman	− 0.048	0.118	0.172	0.186	− 0.052	0.171
	Sig	0.756	0.444	0.264	0.227	0.736	0.268
Δ Schwab and England ADL OFF^a^	Spearman	**− 0.399**	**0.302**	**0.302**	**0.426**	− 0.207	0.259
	Sig	**0.007**	**0.046**	**0.046**	**0.004**	0.178	0.089
Δ IPAQ	Pearson	0.002	− 0.028	− 0.228	− 0.075	0.675**	− 0.114
	Sig	0.988	0.855	0.137	0.631	** < 0.001**	0.461

Bold values are significant

*MDS-UPDRS*  Movement Disorder Society—Unified Parkinson’s Disease Rating Scale *PIGD*  postural instability and gait disorder, *ADL*  activities of daily living, *IPAQ*  International Physical Activity Questionnaire, *MMSE*  Mini-Mental State Examination, *NMSS* Non-Motor Symptoms Scale, *BDI*  Beck Depression Inventory, *PDQ_39*  Parkinson’s Disease Questionnaire-39

^*^p value is significant if < 0.05

^**^After Bonferroni correction, *p* value is significant if < 0.003

PIGD progression was directly correlated to LDL level (rho = 322, *p* = 0.043). Motor complications progression was moderately correlated to triglycerides level (rho = 0.313, *p* = 0.049). These correlations were non-significant after Bonferroni correction. No other significant correlations with other test and MRI findings were detected (Table 5).

**Table 5:** Correlations between progression of motor and physical activity with laboratory and imaging characteristics

Absolute change between baseline and 1-year follow-up		HbA1c%^a^	Uric acid (mg/dL)	Cholesterol (mg/dL)	Triglycerides mg/dL)^a^	LDL (mg/dL)	HDL (mg/dL)^a^	Fazekas total^a^	Scheltens total^a^
Δ MDS-UPDRS total score OFF	Pearson	0.027	0.102	0.226	− 0.056	0.295	− 0.113	− 0.084	− 0.003
	Sig	0.865	0.532	0.161	0.731	0.065	0.498	0.592	0.985
Δ Axial OFF^a^	Spearman	0.000	0.209	0.133	0.056	0.256	− 0.064	0.002	0.028
	Sig	0.998	0.196	0.415	0.733	0.110	0.701	0.990	0.859
Δ PIGD OFF^a^	Spearman	− 0.194	0.276	0.171	0.230	**0.322**	− 0.068	− 0.089	0.004
	Sig	0.224	0.084	0.292	0.154	**0.043**	0.685	0.571	0.978
Δ Motor complication total score^a^	Spearman	− 0.217	− 0.111	0.065	**0.313**	0.013	− 0.050	− 0.146	− 0.059
	Sig	0.172	0.495	0.691	**0.049**	0.939	0.766	0.350	0.704
Δ Hoehn and Yahr OFF^a^	Spearman	0.198	0.148	0.122	− 0.151	0.139	− 0.005	0.041	0.105
	Sig	0.215	0.362	0.452	0.351	0.393	0.976	0.795	0.496
Δ Schwab and England ADL OFF^a^	Spearman	0.113	0.020	− 0.113	− 0.102	− 0.086	0.051	− 0.019	0.096
	Sig	0.482	0.902	0.487	0.532	0.598	0.760	0.901	0.537
Δ IPAQ	Pearson	0.066	0.009	− 0.014	0.027	0.018	0.023	0.086	− 0.136
	Sig	0.683	0.958	0.933	0.867	0.912	0.891	0.582	0.379

Bold values are significant

*LDL*  low-density lipoproteins, *HDL* = high-density lipoproteins, *MDS-UPDRS*  Movement Disorder Society—Unified Parkinson’s Disease Rating Scale *PIGD* = postural instability and gait disorder, *ADL*  activities of daily living, *IPAQ*  International Physical Activity Questionnaire

^*^p value is significant if < 0.05

^**^After Bonferroni correction, *p* value is significant if < 0.003

Predictors of clinical progression were assessed by linear regression from the following variables: DOI, total MDS-UPDRS, MDS-UPDRS III, tremor subscore, PIGD subscore, Hoehn and Yahr stage, LEDD, total non-motor scale score, BDI, TUG, (10-MWT) comfortable speed, NFOG-Q, Fazekas total score, uric acid. Using linear regression, there was no predictors of clinical progression among the variables used.

## Discussion

The rate of progression of PD differs widely among individual patients [26]. Therefore, many studies are required to determine progression of PD and its predictors. The current study investigated disease progression within 1 year and its potential clinical and laboratory predictors in a cohort of patients with PD in an under-investigated population. Despite of the short follow-up of the current study, identifying disease progression within 1 year enables comparing annual progression with other populations. Additionally, the annual motor progression was suggested as a predictor of long-term disease progression [27].

The current study confirmed other previous observations. Significant motor progression from baseline was first detected at 1-year follow-up consistent with previous studies [28]. Furthermore, variability of motor progression among patients with PD was also observed similar to previous studies [2, 28].

Remarkably, the current study showed a high rate of motor progression compared to previous studies [2, 27, 29–31]. The mean difference of MDS-UPDRS III was − 11.83, while the rate of progression was 26.93% within 1 year. Schrag and his colleagues showed mean annual progression rates of motor impairment and disability ranged from 2.4 to 7.4%. This could be explained by the lower age of onset and severity of the current cohort, as motor progression decrease with advancing disease [2]. Moreover, the presence of medical comorbidities in about half of recruited patients (46.7%) also could explain this higher rate of progression [32].

Remarkably, most of the 1-year follow-ups in the current study were performed during the COVID-19 pandemic. Therefore, these special circumstances may be another important factor for that fast progression which showed agreement with a study done in 2021 in India. The study showed that the most common motor symptom to worsen was motor slowness which was seen in 69.2% of cases followed by tremors, rigidity and gait freezing [33]. Additionally, patients with PD reported worse mental health, quality of life, and physical inactivity during this pandemic [34]. Patients with PD are more vulnerable to recent stressors as a dopamine depleted brain leads to reduced coping mechanisms to stress. Moreover, chronic stress may lead to increased striatonigral dopaminergic neuron loss via oxidative damage to the cell membrane, inflammatory and regulatory T-cell dysfunction, thus leading to worsening of motor symptoms, also it leads to greater activation of locus coeruleus–noradrenergic pathways and may worsen the resting tremor in PD [35, 36].

In addition to individual variability, motor progression of PD is variable between patients and disease subgroups. The Schwab and England ADL progression was more significant in patients with advanced stages of PD compared to wild PD, and this was consistent with previous studies which reported that mobility, activities of daily living, communication, and stigma were more progressed in advanced PD patients [37].

There was no significant difference in progression in either males or females. Similarly, Reinoso and his colleagues described a similar motor progression in males and females over 1 year, but significant differences were detected in longer periods [30]. More recently, another study with a large cohort confirmed similar rate of progression of males and females [38]. Inconsistently, Chahine and his colleagues reported male gender as a predictor for motor progression [27] and a recent meta-analysis suggested that females progress more slowly regarding ADL impairment and develop dyskinesia early [39], implying the need for further studies.

Minimal differences in motor progression were detected between TD and non-TD subtypes including disease severity (Hoehn and Yahr) and physical activity, similar to previous studies. Reinoso and his colleagues reported higher motor progression with akinetic rigid subtype [30]. Similarly, another study reported subtle differences between TD and PIGD subtypes during 4-year observation, despite more severe manifestations at baseline [40]. A previous study showed more rapid motor progression with PIGD versus TD, but without predictive value [5].

Comparing early-onset and late-onset PD at baseline showed no significant difference in motor progression. This is inconsistent with a previous study that found slower progression in EOPD and suggests that the preclinical interval in this group is longer [41]. Another study showed a similar progression rate in the first 4 years post-diagnosis, followed by more rapid progression in the older group [30]. Younger age and shorter duration of our cohort, small sample size and short duration of follow-up may explain these differences.

Predictors of motor progression were variable among previous studies and included male gender, higher baseline motor score, male sex, and increased age, akinetic rigid subtype, cognitive impairment, nondopaminergic symptoms in addition to genetic factors and CSF biomarkers [3, 30, 42]. The current study showed significant associations between motor progression over 1 year and baseline scores, especially Schwab and England ADL progression and gait parameters. However, this study did not detect significant predictors of motor progression over 1 year that could be explained by small sample size, short duration of follow-up and different subtypes of PD included in this cohort. Furthermore, this was consistent with previous studies which showed conflicting results in detecting prognostic factors of PD progression due to short duration of follow-up and variable methods used [43]. Therefore, longer follow-up and larger cohorts are warranted.

A recent population-based study showed the influence of lifestyle factors including physical activity on PD progression [44]. Therefore, the role of physical activity was investigated in this study and showed significant association with worsening of Schwab and England ADL and physical activity, denoting its potential prognostic value and the importance of improving the lifestyle of people with PD [45]. Moreover, baseline NMS showed associated with progression of total MDS-UPDRS, PIGD, Schwab and England ADL and axial scores. Similarly, Ayala and his colleagues reported the NMS as a predictor of disease progression over 3 years [46]. Remarkably, the current cohort is characterized by younger age and age of onset, shorter disease duration, better baseline cognition, that affect study outcome and interpretation.

Previous studies linked WMH severity or volume to motor, gait and cognitive progression of PD, implying its usefulness as a prognostic marker [47–49]. The current study showed variability of WMH in size in agreement with previous studies [50], yet these changes could not predict progression over 1 year. Inconsistency with other studies may be attributed to different methodology, a small number of patients, using low-resolution MRI, short follow-up and low WMH load of most of the recruited patients. Therefore, further MRI studies are warranted to evaluate short prognostic values of WMH.

Baseline lipid profile was not correlated to motor progression of PD in the current study in agreement with previous studies, which failed to find a significant linkage between lipid profile abnormalities and PD progression [51, 52]. Triglyceride level was correlated to progression of motor complications in this study, which is inconsistent with previous studies demonstrating no significant association between triglycerides and motor or cognitive progression [51]. However, previous studies revealed controversy regarding the association between lipid profile and PD progression [53]. Serum uric acid was not correlated to PD progression in this cohort, in contrast to previous studies which reported that low levels of serum uric acid associated with a high risk of PD progression and worsening of UPDRS scores [54, 55]. Moreover, HbA1c was not correlated to PD progression in this study in agreement with the uncertainty about the role of HbA1c to predict PD progression in previous studies [56]. Younger age, shorter duration of our cohort, small sample size and short duration of follow-up should be considered when interpreting the current findings.

Although investigating the baseline predictors (clinical and biochemical) of PD in a follow-up study over 1 year is important, larger sample size with a longer duration is required for more convenient analysis. Furthermore, including more biochemical biomarkers and advanced neuroimaging are required. Moreover, the COVID-19 pandemic impacted the recruitment of the patients, in addition to its possible effect on disease progression. Also, assessment of therapy adherence among PD patients is required especially during COVID-19 pandemic.

## Conclusion

The current study demonstrated the motor progression over 1 year in an under-investigated population that showed high annual worsening. It showed significant associations between motor progression over 1 year and baseline scores, especially Schwab and England ADL progression, and gait parameters, but without predictive values. Remarkably, it highlighted the progression of physical activity. However, further longitudinal studies with a larger number are warranted to detect and confirm predictors for short-term progression.

## Supplementary Information

**Additional file 1: Table S1.** Education, comorbidities, laboratory results, brain imaging, and Hoehn and Yahr stagging of PD patients at baseline. **Table S2.** Percentage change of motor and physical activity over 6 months and 1-year follow-up. **Table S3.** Comparison between mild PD vs advanced-moderate PD regarding baseline characteristics and motor progression. **Table S4.** Gender comparison regarding baseline characteristics and motor progression. **Table S5.** Comparison between Tremor Dominant and non-TD patients regarding baseline characteristics and motor progression. **Table S6.** Comparison between late onset vs early onset PD regarding baseline characteristics and motor progression. **Table S7.** Correlations between progression of motor subscores with baseline demographic, clinical, Lab and imaging characteristics.

## Data Availability

All data generated or analyzed during the current study are included in this published article (and its Additional information files).

## Abbreviations

ADL: Activities of daily living
AOO: Age of onset
BBS: Berg Balance Scale
BDI: Beck Depression Inventory
COVID-19: Coronavirus disease of 2019
CSF: Cerebrospinal fluid
DOI: Duration of illness
EOPD: Early-onset PD
HDL: High-density lipoprotein
IPAQ: International Physical Activity Questionnaire
LDL: Low-density lipoprotein
LEDD: Levodopa equivalent daily dose
LOPD: Late-onset PD
MDS: Movement Disorder Society
MMSE: Mini-Mental State Examination
MRI: Magnetic resonance imaging
NFOG-Q: New Freezing Of Gait Questionnaire
NMSS: Non-Motor Symptoms Scale
PD: Parkinson’s disease
PDQ-39: PD Questionnaire-39
QoL: Quality of life
RBD: Rapid eye movement sleep behavior disorder
RMANOVA: Repeated measures ANOVA
TD: Tremor dominant
TUG: Time Up and Go Test
UPDRS: Unified Parkinson’s Disease Rating Scale
WMHL: White matter hyperintensities lesions
10-MWT: 10-Meter Walking Test
