# Supplementary material for: Baseline predictors of progression of Parkinson’s disease in a sample of Egyptian patients: clinical and biochemical
Source: Egypt J Neurol Psychiatr Neurosurg. 2022 Jan 15;58(1):9. doi: 10.1186/s41983-022-00445-1 (PMC8760567; doi:10.1186/s41983-022-00445-1)
Supplement: Supplementary file 1 — Additional file 1: Table S1. Education, comorbidities, laboratory results, brain imaging, and Hoehn and Yahr stagging of PD patients at baseline. Table S2. Percentage change of motor and physical activity over 6 months and 1-year follow-up. Table S3. Comparison between mild PD vs advanced-moderate PD regarding baseline characteristics and motor progression. Table S4. Gender comparison regarding baseline characteristics and motor progression. Table S5. Comparison between Tremor Dominant and non-TD patients regarding baseline characteristics and motor progression. Table S6. Comparison between late onset vs early onset PD regarding baseline characteristics and motor progression. Table S7. Correlations between progression of motor subscores with baseline demographic, clinical, Lab and imaging characteristics. [file 41983_2022_445_MOESM1_ESM.docx]

**Additional tables**

**Table S1:** **Education , comorbidities, laboratory results , brain imaging, and Hoehn and Yahr stagging of PD patients at baseline:**

|  | | | **Mean (SD) /Frequency (%)** | **Range** |
| --- | --- | --- | --- | --- |
|  |  |  |  |  |
| **LEDD** | | | 596.36 (334.37) | 0-1550 |
| **Education (years)** | | | 6.08 (5.72) | 0-18 |
| **Education** | **Illiterate** | | 14 (31.1%) | |
|  | **Read and write** | | 8 (17.8%) | |
|  | **6 years education** | | 4 (8.9%) | |
|  | **9 years education** | | 8 (17.8%) | |
|  | **High school** | | 7 (15.6%) | |
|  | **University graduated** | | 4 (8.9%) | |
| **Functioning** | **Not- functioning** | | 7 (15.6%) | |
|  | **Functioning** | | 28 (62.2%) | |
|  | **Functioning and retired** | | 10 (22.2%) | |
| **Comorbidities number** | **No** | | 24 (53.3%) | |
|  | **One** | | 13 (28.9%) | |
|  | **Two** | | 5 (11.1%) | |
|  | **Three** | | 3 (6.7%) | |
| **Diabetes mellitus** | | | 8 (17.8%) | |
| **Hypertension** | | | 13 (28.9%) | |
| **Ischemic heart disease** | | | 7 (15.6%) | |
| **Hyperlipidemia** | | | 2 (4.4%) | |
| **Hepatitis C virus** | | | 2 (4.4%) | |
| **Smoking** | **Smoker** | | 6 (13.3%) | |
|  | **Ex- smoker** | | 11 (24.4%) | |
| **Substance abuse** | | | 3 (6.7%) | |
| **Consanguinity** | | | 14 (31.1%) | |
| **Family History of Neuropsychiatric illness** | **No FH of psychiatric illness** | | 33 (73.3%) | |
|  | **FH of PD** | | 9 (20%) | |
|  | **FH of psychiatric illness** | | 2 (4.4%) | |
|  | **FH of dementia** | | 1 (2.2%) | |
| **Tremors OFF** | | | 12.80(7.47) | 0-27 |
| **Bradykinesia OFF** | | | 15.93(7.57) | 1-34 |
| **Rigidity OFF** | | | 8.91(4.15) | 3-20 |
| **Axial OFF** | | | 13.93(6.88) | 4-30 |
| **PIGD OFF** | | | 8.55(5.72) | 1-20 |
| **Motor complication total score** | | | 4.93(4.48) | 0-18 |
| **Hoehn and Yahr stages** | | **Stage 1.5** | 6 (13.3%) | |
|  |  | **Stage 2** | 7 (15.6%) | |
|  |  | **Stage 2.5** | 21 (46.7%) | |
|  |  | **Stage 3** | 6 (13.3 %) | |
|  |  | **Stage 4** | 5 (11.1%) | |
| **Hoehn and Yahr severity** | | **Mild (stage ≤ 2.5)** | 34 (75.6%) | |
|  |  | **Moderate (stage = 3)** | 6 (13.3%) | |
|  |  | **Severe (stage >3)** | 5 (11.1%) | |
| **Laboratory results** | **HbA1c %** | | 5.96 (0.99) | 4.4-9.8 |
|  | **Uric acid (mg/dL)** | | 5.81(0.69) | 4.4-7.5 |
|  | **Cholesterol (mg/dL)** | | 177.65 (46.31) | 90-313 |
|  | **Triglycerides (mg/dL)** | | 120.78 (45.87) | 60-294 |
|  | **LDL (mg/dL)** | | 107.92 (30.70) | 44-181 |
|  | **HDL (mg/dL)** | | 40.42 (10.28) | 29-71 |
| **Shelton total score** | | | 2.84 (3.34) | 0-14 |
| **Periventricular** | **0=No abnormality** | | 18 (40.9%) | |
|  | **1=lesions less than 3 mm, ≤5 lesions** | | 15 (34.1%) | |
|  | **3=lesions 4 to 10 mm, ≤5 lesions** | | 4 (9.1%) | |
|  | **4=lesions 4 to 10 mm, >6 lesions** | | 1(2.3%) | |
|  | **6=confluent lesions** | | 6 (13.6%) | |
| **Deep White Matter** | **0=No abnormality** | | 16 (36.4%) | |
|  | **1=lesions less than 3 mm, ≤5 lesions** | | 18 (40.9%) | |
|  | **2=lesions < 3 mm, more than 6 lesions** | | 1(2.3%) | |
|  | **3=lesions 4 to 10 mm, ≤5 lesions** | | 7 (15.9%) | |
|  | **4=lesions 4 to 10 mm, but >6 lesions** | | 1(2.3%) | |
|  | **6=confluent lesions** | | 1 (2.3%) | |
| **Basal Ganglion** | **0=No abnormality** | | 41 (93.2%) | |
|  | **1=lesions less than 3 mm, ≤5 lesions** | | 3 (6.8%) | |
| **Infratentorial** | **0=No abnormality** | | 40 (90.9%) | |
|  | **1=lesions less than 3 mm, ≤5 lesions** | | 4 (9.1%) | |
| **Fazekas-Total** | **0=No or single punctate lesion** | | 16 (37.2%) | |
|  | **1=Multiple punctate lesions** | | 22 (51.2%) | |
|  | **2=Beginning confluence of lesions (bridging)** | | 5 (11.6%) | |

LEDD= Levodopa equivalent daily dose, LDL= Low density lipoprotein, HDL= High density lipoprotein, PIGD= postural instability and gait disorder.

**Table S2:** **percentage change of motor and physical activity over 6 months and 1**- **year follow up:**

|  | **Percentage Change 1st 6 months** | **Percentage Change 2nd 6 months** | **Percentage Change total** |
| --- | --- | --- | --- |
|  | **Mean (SD)** | **Mean (SD)** | **Mean (SD)** |
| **MDS-UPDRS total score OFF** | 8.54(12.67) | 23.50(11.38) | 33.88(19.80) |
| **MDS-UPDRS- I** | 20.11(48.66) | 42.30(35.59) | 72.0(104.46) |
| **MDS-UPDRS -II** | 21.65(38.0) | 24.94(25.45) | 53.10(54.45) |
| **MDS-UPDRS -III OFF** | 4.33(10.68) | 22.19(14.05) | 26.93(17.24) |
| **Tremor OFF** | 7.44(29.16) | 11.33(28.82) | 19.73(48.50) |
| **Bradykinesia OFF** | 12.49(47.70) | 29.02(23.82) | 42.96(60.45) |
| **Rigidity OFF** | 6.65(17.79) | 21.55(28.08) | 27.77 (32.40) |
| **Axial OFF^ꭝ^** | 10.60(19.01) | 27.14(22.52) | 39.97 (33.28) |
| **PIGD OFF^ꭝ^** | 14.29(41.73) | 29.43(47.17) | 47.77(79.06) |
| **Motor complication total score^ꭝ^** | 6.73(21.40) | 20.58(26.48) | 24.26 (39.21) |
| **Hoehn and Yahr OFF^ꭝ^** | 4.07(11.69) | 14.89(16.94) | 18.78 (19.81) |
| **Schwab and England ADL OFF^ꭝ^** | -7.12(9.40) | -10.97(7.85) | -17.19 (12.55) |
| **IPAQ** | -22.48(23.99) | -27.54(13.67) | -43.56 (20.18) |

MDS-UPDRS= movement disorder society – unified Parkinson’s disease rating scale PIGD= postural instability and gait disorder, ADL = activities of daily living, IPAQ = international physical activity questionnaire, RMANOVA=Repeated Measures ANOVA

**Table S3 Comparison between mild PD vs advanced-moderate PD regarding baseline characteristics and motor progression :**

|  | **Mild PD**  **(No.=34)** | **Advanced and moderate PD**  **(No.=11)** | **Mann Whitney** | |
| --- | --- | --- | --- | --- |
|  | **Mean (SD)** | **Mean (SD)** | **z** | **P** |
| **Age** | 57.10 (7.87) | 54.45 (13.09) | -1.12 | 0.26 |
| **AOO** | 52.47 (8) | 47.77 (13.47) | -1.48 | 0.14 |
| **DOI** | 4.26 (3) | 6.86 (2.61) | -2.65 | **0.01** |
| **LEDD** | 535.68 (276.20) | 783.91 (434.63) | -1.85 | 0.06 |
| **Years of education** | 6.74 (5.87) | 4.09 (4.97) | -1.28 | 0.20 |
| **MDS-UPDRS total score OFF baseline** | 70.65 (23.12) | 116.82 (34.06) | -3.57 | **<0.001** |
| **MDS-UPDRS- I OFF baseline** | 13.65 (5.38) | 20.64 (9.73) | -2.44 | **0.01** |
| **MDS-UPDRS- II OFF baseline** | 15.38 (8.40) | 27.73 (9.37) | -3.49 | **<0.001** |
| **MDS-UPDRS-III OFF baseline** | 41.44 (15.03) | 68.45 (18.51) | -3.58 | **<0.001** |
| **Tremors OFF baseline** | 12.12 (7.10) | 15.18 (9.04) | -1.16 | 0.24 |
| **Bradykinesia OFF baseline** | 13.82 (6.05) | 23.18 (7.67) | -3.21 | **<0.001** |
| **Rigidity OFF baseline** | 7.85 (3.69) | 11.91 (4.01) | -2.80 | **0.01** |
| **Axial OFF baseline** | 10.97 (4.38) | 22.64 (5.41) | -4.46 | **<0.001** |
| **PIGD OFF baseline** | 6.15 (4.13) | 15.45 (3.91) | -4.27 | **<0.001** |
| **Motor complication total score baseline** | 3.35 (3.26) | 9.36 (4.84) | -3.55 | **<0.001** |
| **Schwab and England ADL OFF baseline** | 81.18 (8.08) | 60.91 (13.75) | -4.26 | **<0.001** |
| **TUG OFF baseline** | 14.34 (10.60) | 33.25 (22.09) | -3.88 | **<0.001** |
| **(10- MWT) comfortable speed OFF baseline** | 0.86 (0.28) | 0.49 (0.29) | -3.22 | **0.001** |
| **10- MWT) maximum speed OFF baseline** | 1.16 (0.36) | 0.70 (0.33) | -3.20 | **0.001** |
| **BBS OFF baseline** | 49.71 (5.30) | 33.91 (11.113) | -4.06 | **<0.001** |
| **NFOG-Q OFF baseline** | 7.12 (8.29) | 20.27 (8.88) | -3.78 | **<0.001** |
| **IPAQ baseline** | 2273.18 (745.50) | 1447.82 (728.90) | -2.91 | **<0.001** |
| **MMSE baseline** | 27.15 (2.24) | 24.36 (4.50) | -1.79 | 0.07 |
| **NMSS total score baseline** | 56.44 (38.43) | 78.27 (55.99) | -1.36 | 0.17 |
| **BDI baseline** | 15.35 (7.92) | 24.09 (12.31) | -2.46 | **0.01** |
| **PDQ-39 baseline** | 32.28 (15.57) | 51.53 (19.88) | -2.96 | **<0.001** |
| **HbA1c %** | 6.10 (1.08) | 5.53 (0.52) | -1.98 | 0.05 |
| **Uric acid (mg/dL)** | 5.75 (0.67) | 6.02 (0.74) | -1.02 | 0.31 |
| **Cholesterol (mg/dL)** | 171.70 (48.62) | 195.50 (34.70) | -1.63 | 0.10 |
| **Triglycerides (mg/dL)** | 125.63 (49.85) | 106.20 (28.20) | -1.09 | 0.27 |
| **LDL (mg/dL)** | 103.40 (30.68) | 121.46 (27.96) | -1.31 | 0.19 |
| **HDL (mg/dL)** | 38.50 (7.97) | 45.80 (14.15) | -1.40 | 0.16 |
| **Shelten total score** | 3.09 (3.56) | 2.09 (2.63) | -0.67 | 0.50 |
| **Fazekas total score** | 0.76 (0.66) | 0.70 (0.67) | -0.24 | 0.81 |
| **Clinical progression** | | | | |
| **Δ MDS-UPDRS-OFF total score** | 24.85 (11.79) | 21.91 (13.92) | -0.76 | 0.45 |
| **Δ MDS-UPDRS- I OFF** | 11.85 (7.08) | 11.00 (6.96) | -1.80 | 0.07 |
| **Δ MDS-UPDRS- II OFF** | -875.03 (527.39) | -863.09 (612.23) | -0.08 | 0.93 |
| **Δ MDS-UPDRS-III OFF** | -10.91 (7.65) | -17.27 (7.86) | -0.35 | 0.72 |
| **Δ Tremors** | 1.82 (2.81) | 1.91 (2.17) | -0.20 | 0.84 |
| **Δ Rigidity** | 1.64 (1.78) | 1.91 (1.38) | -0.24 | 0.81 |
| **Δ Axial** | 4.39 (3.09) | 4.00 (3.55) | -0.89 | 0.37 |
| **Δ PIGD** | 2.15 (3.04) | 1.91 (2.95) | -0.07 | 0.95 |
| **Δ Motor complication total score** | 2.24 (2.62) | 1.18 (2.23) | -1.37 | 0.17 |
| **Δ Schwab and England ADL OFF** | -10.91 (7.65) | -17.27 (7.86) | -2.50 | **0.01** |
| **Δ IPAQ** | -875.03 (527.39) | -863.09 (612.23) | -0.30 | 0.77 |

AOO = age of onset, DOI= duration of illness, LEDD= Levodopa equivalent daily dose, LDL= Low density lipoprotein, HDL= High density lipoprotein, MDS-UPDRS= movement disorder society – unified Parkinson’s disease rating scale PIGD= postural instability and gait disorder,ADL = activities of daily living, TUG= time up and go test, 10 MWT= 10 meter walking test , BBS = berg balance scale , NFOG-Q = new freezing of gait questionnaire, IPAQ = international physical activity questionnaire, MMSE=mini mental state examination ,NMSS = non-motor symptoms scale ,BDI = beck depression inventory ,PDQ_39= Parkinson’s disease questionnaire-39.

* p value is significant if<0.05

**Table S4:** **Gender comparison regarding baseline characteristics and motor progression :**

|  | **Male**  **(No.=34)** | **Female**  **(No.=11)** | **Mann Whitney** | |
| --- | --- | --- | --- | --- |
|  | **Mean (SD)** | **Mean (SD)** | **z** | **p** |
| **Age** | 55.96 (8.75) | 58.00 (11.20) | -1.07 | 0.28 |
| **AOO** | 50.88 (9.00) | 52.68 (11.87) | -1.02 | 0.31 |
| **DOI** | 4.73 (3.19) | 5.43 (2.86) | -0.98 | 0.33 |
| **LEDD** | 592.97 (336.34) | 606.82 (344.07) | -0.49 | 0.62 |
| **Years of education** | 6.47(5.86) | 4.91 (5.38) | -0.77 | 0.44 |
| **MDS-UPDRS total score OFF baseline** | 78.32 (30.62) | 92.82 (37.92) | -1.11 | 0.27 |
| **MDS-UPDRS -I baseline** | 14.53 (6.70) | 17.91 (8.55) | -0.85 | 0.40 |
| **MDS-UPDRS -II baseline** | 17.09 (9.55) | 22.45 (11.06) | -1.39 | 0.16 |
| **MDS-UPDRS-III OFF baseline** | 46.53 (19.12) | 52.45 (21.52) | -0.85 | 0.40 |
| **Tremors OFF baseline** | 13.15 (7.87) | 12.45 (6.46) | -0.21 | 0.83 |
| **Bradykinesia OFF baseline** | 15.59 (7.54) | 17.73 (7.80) | -0.86 | 0.39 |
| **Rigidity OFF baseline** | 8.85 (4.17) | 8.82 (4.17) | -0.08 | 0.94 |
| **Axial OFF baseline** | 12.74 (6.17) | 17.18 (7.97) | -1.75 | 0.08 |
| **PIGD OFF baseline** | 7.24 (5.14) | 12.09 (6.06) | -2.20 | **0.03*** |
| **Motor complication total score baseline** | 4.38 (4.57) | 6.18 (4.14) | -1.61 | 0.11 |
| **Hoehn and Yahr OFF baseline** | 2.46 (0.63) | 2.73 (0.82) | -1.36 | 0.17 |
| **Schwab and England ADL OFF baseline** | 77.94 (12.25) | 70.91(14.46) | -1.55 | 0.12 |
| **TUG OFF baseline** | 15.22 (10.65) | 30.53 (24.23) | -2.17 | **0.03*** |
| **(10-MWT) comfortable speed OFF baseline** | 0.84 (0.29) | 0.56 (0.35) | -2.25 | **0.02*** |
| **(10-MWT) maximum speed OFF baseline** | 1.13 (0.37) | 0.75 (0.47) | -2.18 | **0.03*** |
| **BBS OFF baseline** | 47.35 (8.57) | 41.18 (12.22) | -1.55 | 0.12 |
| **NFOG-Q OFF baseline** | 8.47 (9.57) | 16.09 (9.96) | -2.21 | **0.03*** |
| **IPAQ baseline** | 2148.19(780.00) | 1834.14 (917.49) | -0.87 | 0.38 |
| **MMSE baseline** | 26.68 (3.00) | 25.82 (3.60) | -0.65 | 0.51 |
| **NMSS total score baseline** | 59.15 (46.73) | 69.91 (33.22) | -1.37 | 0.17 |
| **BDI baseline** | 15.94 (8.72) | 22.27 (11.72) | -1.60 | 0.11 |
| **PDQ-39 baseline** | 33.69 (16.82) | 47.17 (20.45) | -1.74 | 0.08 |
| **HbA1c %** | 5.90 (0.88) | 6.17 (1.37) | -0.06 | 0.95 |
| **Uric acid (mg/dL)** | 5.83 (0.65) | 5.78 (0.87) | -0.46 | 0.65 |
| **Cholesterol (mg/dL)** | 177.19 (45.59) | 179.22 (51.56) | -0.24 | 0.81 |
| **Triglycerides (mg/dL)** | 123.77 (48.81) | 110.44 (34.16) | -0.50 | 0.62 |
| **LDL (mg/dL)** | 107.81 (28.41) | 108.29 (39.63) | -0.40 | 0.69 |
| **HDL (mg/dL)** | 37.97 (7.21) | 49.63 (14.85) | -2.28 | **0.02*** |
| **Shelten total score** | 2.58 (3.36) | 3.64 (3.32) | -1.17 | 0.24 |
| **Fazekas total score** | 0.729 (0.63) | 0.82 (0.75) | -0.35 | 0.72 |
| **Clinical progression** | | | | |
| **Δ MDS-UPDRS total score OFF** | 25.45 (13.13) | 20.09 (7.89) | -1.248 | 0.212 |
| **Δ MDS-UPDRS-I** | 6.73 (4.73) | 5.00 (3.58) | -1.063 | 0.288 |
| **Δ MDS-UPDRS-II** | 7.24 (5.61) | 4.45 (4.61) | -1.332 | 0.183 |
| **Δ MDS-UPDRS-III OFF** | 12.03(7.64) | 10.45 (4.52) | -0.598 | 0.550 |
| **Δ Tremors OFF** | 1.91 (2.65) | 1.63 (2.73) | -0.697 | 0.486 |
| **Δ Bradykinesia OFF** | 4.82 (3.36) | 4.63 (3.26) | -0.369 | 0.712 |
| **Δ Rigidity OFF** | 1.61 (1.73) | 2.00 (1.55) | -0.402 | 0.688 |
| **Δ Axial OFF** | 4.64 (3.12) | 3.27 (3.26) | -1.340 | 0.180 |
| **Δ PIGD OFF** | 2.45 (3.10) | 1.00 (2.41) | -1.379 | 0.168 |
| **Δ Motor complication total score** | 2.00(2.63) | 1.91 (2.39) | -0.014 | 0.989 |
| **Δ Hoehn and Yahr OFF** | 0.41 (0.44) | 0.64 (0.60 | -1.108 | 0.268 |
| **Δ Schwab and England ADL OFF** | -11.52 (7.95) | -15.45 (8.20) | -1.499 | 0.134 |
| **Δ IPAQ** | -850.95 (551.85) | -935.32 (533.49) | -0.867 | 0.386 |

AOO = age of onset, DOI= duration of illness, LEDD= Levodopa equivalent daily dose, LDL= Low density lipoprotein, HDL= High density lipoprotein, MDS-UPDRS= movement disorder society – unified Parkinson’s disease rating scale PIGD= postural instability and gait disorder, ADL = activities of daily living, TUG= time up and go test, 10 MWT= 10 meter walking test, BBS = berg balance scale, NFOG-Q = new freezing of gait , IPAQ = international physical activity questionnaire, MMSE=mini mental state examination, NMSS = non-motor symptoms scale ,BDI = beck depression inventory, PDQ_39= Parkinson’s disease questionnaire-39.

* p value is significant if<0.05

**Table S5: Comparison between Tremor Dominant and non -TD patients regarding baseline characteristics and motor progression :**

|  | **PD subtypes** | | **Mann-Whitney** | |
| --- | --- | --- | --- | --- |
|  | **TD**  **(No.=34)** | **Non-TD**  **(No.=11)** |  |  |
|  | **Mean (SD)** | **Mean (SD)** | **z** | **p** |
| **Age** | 58.02 (7.29) | 51.59 (13.11) | -1.468 | 0.142 |
| **AOO** | 53.43 (7.69) | 44.82 (12.43) | -1.996 | **0.046*** |
| **DOI** | 4.26 (2.67) | 6.86 (3.61) | -2.068 | **0.039*** |
| **LEDD** | 551.79 (325.88) | 734.09 (337.32) | -1.363 | 0.173 |
| **Years of education** | 5.06 (5.54) | 9.27 (5.31) | -2.175 | **0.030*** |
| **MDS-UPDRS total score OFF baseline** | 81.85 (33.92) | 81.91 (30.14) | -0.066 | 0.947 |
| **MDS-UPDRS- I baseline** | 14.71 (6.82) | 17.36 (8.45) | -0.702 | 0.483 |
| **MDS-UPDRS -II baseline** | 17.29 (10.67) | 21.82 (7.37) | -1.587 | 0.112 |
| **MDS-UPDRS-III OFF baseline** | 49.68 (20.08) | 42.73 (7.37) | -1.295 | 0.195 |
| **Tremors OFF baseline** | 15.53 (6.53) | 5.09 (3.91) | -4.140 | **<0.001*** |
| **Bradykinesia OFF baseline** | 16.50 (7.62) | 14.91 (7.67) | -0.820 | 0.412 |
| **Rigidity OFF baseline** | 8.88 (4.10) | 8.73 (4.41) | -0.119 | 0.905 |
| **Axial OFF baseline** | 12.85 (6.50) | 16.82 (7.28) | -1.814 | 0.070 |
| **PIGD OFF baseline** | 7.09 (5.17) | 12.55 (5.54) | -2.788 | **0.005*** |
| **Motor complication total score baseline** | 4.15 (4.05) | 6.91 (13.62) | -1.733 | 0.083 |
| **Hoehn and Yahr OFF baseline** | 2.49 (0.65) | 2.64 (7.37) | -0.378 | 0.705 |
| **Schwab and England ADL OFF baseline** | 76.18 (13.03) | 76.36 (13.62) | -0.041 | 0.967 |
| **TUG OFF baseline** | 14.82 (8.73) | 31.79 (25.79) | -1.480 | 0.139 |
| **(10- MWT) comfortable speed OFF baseline** | 0.82 (0.31) | 0.62 (0.36) | -2.060 | **0.039*** |
| **(10- MWT) maximum speed OFF baseline** | 1.11 (0.40) | 0.79 (0.43) | -0.932 | 0.351 |
| **BBS OFF baseline** | 46.74 (9.95) | 43.09 (9.27) | -1.443 | 0.149 |
| **NFOG-Q OFF baseline** | 7.29 (8.88) | 19.73 (7.81) | -3.394 | **0.001*** |
| **IPAQ baseline** | 2020.44 (735.02) | 2229 (1054.75) | -0.344 | 0.731 |
| **MMSE baseline** | 26.35 (3.33) | 26.82 (2.56) | -0.147 | 0.883 |
| **NMSS total score baseline** | 60.85 (46.66) | 64.64 (34.80) | -0.502 | 0.615 |
| **BDI baseline** | 16.79 (9.05) | 19.64 (12.04) | -0.634 | 0.526 |
| **PDQ39 baseline** | 36.34 (19.37) | 38.99 (16.09) | -2.377 | **0.017*** |
| **HbA1c %** | 6.05 (1.08) | 5.64 (0.57) | -0.932 | 0.351 |
| **Uric acid (mg/dL)** | 5.77 (0.64) | 6.01 (0.89) | -0.289 | 0.772 |
| **Cholesterol (mg/dL)** | 180.25 (45.08) | 167.25 (52.85) | -0.338 | 0.735 |
| **Triglycerides (mg/dL)** | 125.13 (47.81) | 103.38 (34.14) | -1.167 | 0.243 |
| **LDL (mg/dL)** | 108.18 (31.44) | 106.88 (29.57) | -0.271 | 0.787 |
| **HDL (mg/dL)** | 39.20(9.34) | 45 (12.91) | -1.528 | 0.127 |
| **Shelten total score** | 3.03 (3.62) | 2.27 (2.37) | -0.223 | 0.824 |
| **Fazekas total score** | 0.78 (0.65) | 0.64 (0.67) | -0.648 | 0.517 |
| **Clinical Progression** | | | | |
| **Δ MDS-UPDRS- total score OFF** | 24.87 (12.90) | 21.81 (10.26) | -0.380 | 0.704 |
| **Δ MDS- UPDRS- I** | 6.48 (4.56) | 5.73 (4.45) | -0.354 | 0.723 |
| **Δ MDS-UPDRS -II** | 6.97 (5.63) | 5.27 (4.94) | -0.761 | 0.446 |
| **Δ MDS-UPDRS-III OFF** | 11.87 (7.53) | 10.90 (5.20) | -0.258 | 0.796 |
| **Δ Tremors OFF** | 1.60 (2.54) | 2.54 (2.91) | -0.724 | 0.469 |
| **Δ Bradykinesia OFF** | 5.15 (3.50) | 3.63 (2.37) | -1.188 | 0.235 |
| **Δ Rigidity OFF** | 1.82 (1.67) | 1.36 (1.74) | -0.651 | 0.515 |
| **Δ Axial OFF** | 4.33 (2.95) | 4.18 (3.92) | -0.178 | 0.859 |
| **Δ PIGD OFF** | 2.36 (3.18) | 1.27 (2.24) | -0.601 | 0.548 |
| **Δ Motor complication** | 2.12 (2.68) | 1.55 (2.16) | -0.275 | 0.783 |
| **Δ Hoehn and Yahr OFF** | 0.38 (0.45) | 0.73 (0.52) | -2.057 | **0.040*** |
| **Δ Schwab and England ADL OFF** | -11.21 (8.20) | -16.36 (6.74) | -1.925 | 0.054 |
| **Δ IPAQ** | -768.91 (486.04) | -1181.45 (607.17) | -2.507 | **0.012*** |

AOO = age of onset, DOI= duration of illness, LEDD= Levodopa equivalent daily dose, LDL= Low density lipoprotein, HDL= High density lipoprotein, MDS-UPDRS= movement disorder society – unified Parkinson’s disease rating scale PIGD= postural instability and gait disorder, ADL = activities of daily living, TUG= time up and go test, 10 MWT= 10 meter walking test, BBS = berg balance scale, NFOG-Q = new freezing of gait questionnaire, IPAQ = international physical activity questionnaire, MMSE=mini mental state examination, NMSS = non-motor symptoms scale ,BDI = beck depression inventory, PDQ_39= Parkinson’s disease questionnaire-39.

* p value is significant if<0.05

**Table S6: Comparison between late onset vs early onset PD regarding baseline characteristics and motor progression:**

|  | **Late onset PD**  **(No.=30)** | **Early onset PD**  **(No.=15)** | **Mann Whitney** | |  |
| --- | --- | --- | --- | --- | --- |
|  | **Mean (SD)** | **Mean (SD)** | **z** | **p** | |
| **Age** | 60.85 (5.82) | 47.67 (8.85) | -4.63 | **<0.001** | |
| **AOO** | 56.73 (5.68) | 40.50 (6.21) | -5.42 | **<0.001** | |
| **DOI** | 4.17 (2.65) | 6.37 (3.48) | -2.07 | **0.04** | |
| **LEDD** | 541.20 (277.10) | 706.67 (415.60) | -1.51 | 0.13 | |
| **Years of education** | 5.90 (5.76) | 6.47 (5.83) | -0.40 | 0.69 | |
| **MDS-UPDRS- total score OFF baseline** | 79.77 (35.43) | 86.07 (27.06) | -0.96 | 0.34 | |
| **MDS-UPDRS-I OFF baseline** | 15.00 (7.91) | 16.07 (5.86) | -0.57 | 0.57 | |
| **MDS-UPDRS-II OFF baseline** | 17.53 (10.89) | 20.13 (8.31) | -1.06 | 0.29 | |
| **MDS_UPDRS-III OFF baseline** | 47.03 (20.70) | 49.87 (17.88) | -0.57 | 0.57 | |
| **Tremors OFF baseline** | 14.33 (6.77) | 10.27 (8.33) | -1.69 | 0.09 | |
| **Bradykinesia OFF baseline** | 15.20 (7.85) | 17.93 (6.89) | -1.21 | 0.23 | |
| **Rigidity OFF baseline** | 8.63 (4.44) | 9.27 (3.49) | -0.85 | 0.40 | |
| **Axial OFF baseline** | 12.70 (6.62) | 16.07 (6.93) | -1.73 | 0.08 | |
| **PIGD OFF baseline** | 7.33 (5.36) | 10.60 (5.95) | -1.94 | 0.05 | |
| **Motor complication total score baseline** | 3.97 (3.97) | 6.53 (5.10) | -1.64 | 0.10 | |
| **Hoehn and Yahr OFF baseline** | 2.43 (0.69) | 2.70 (0.65) | -1.30 | 0.19 | |
| **Schwab and England ADL OFF baseline** | 76.67 (11.24) | 75.33 (16.42) | -0.10 | 0.92 | |
| **TUG OFF baseline** | 15.33 (10.66) | 26.24 (22.48) | -1.94 | 0.05 | |
| **(10-MWT) comfortable speed OFF baseline** | 0.82 (0.28) | 0.67 (0.39) | -1.11 | 0.27 | |
| **(10-MWT) maximum speed OFF baseline** | 1.11 (0.36) | 0.88 (0.51) | -1.22 | 0.22 | |
| **BBS OFF baseline** | 46.50 (9.63) | 44.53 (10.37) | -0.80 | 0.43 | |
| **NFOG-Q OFF baseline** | 8.53 (9.25) | 13.93 (11.09) | -1.57 | 0.12 | |
| **IPAQ baseline** | 1977.23 (786.25) | 2259.80) (869.69) | -0.84 | 0.40 | |
| **MMSE baseline** | 26.37 (3.24) | 26.67 (3.02) | -0.27 | 0.79 | |
| **NMSS total score baseline** | 64.33 (50.68) | 56.67 (25.35) | -0.10 | 0.92 | |
| **BDI baseline** | 17.30 (11.04) | 17.87 (6.99) | -0.72 | 0.47 | |
| **PDQ-39 baseline** | 36.07 (20.47) | 38.81 (14.15) | -0.99 | 0.32 | |
| **HbA1c %** | 6.18 (1.09) | 5.50 (0.56) | -2.44 | **0.01** | |
| **Uric acid (mg/dL)** | 5.81 (0.73) | 5.82 (0.61) | -0.13 | 0.89 | |
| **Cholesterol (mg/dL)** | 175.68 (50.53) | 182.25 (36.12) | -1.36 | 0.17 | |
| **Triglycerides (mg/dL)** | 118.39 (50.76) | 126.33 (32.99) | -1.27 | 0.20 | |
| **LDL (mg/dL)** | 104.43 (32.38) | 116.05 (25.80) | -1.24 | 0.21 | |
| **HDL (mg/dL)** | 40.96 (11.26) | 39.09 (7.65) | -0.08 | 0.94 | |
| **Shelten total score** | 3.55 (3.70) | 1.47 (2.00) | -2.02 | **0.04** | |
| **Fazekas total score** | 0.86 (0.65) | 0.53 (0.64) | -1.6 | 0.11 | |
| **Clinical Progression** | | | | | |
| **Δ MDS-UPDRS total score OFF** | 24.59 (13.01) | 23.20 (11) | -0.16 | 0.87 | |
| **Δ MDS-UPDRS-I OFF** | 6.28 (4.43) | 6.33 (4.78) | -0.31 | 0.76 | |
| **Δ MDS-UPDRS-II OFF** | 7.21 (5.97) | 5.93 (4.61) | -0.55 | 0.59 | |
| **Δ MDS-UPDRS-III OFF** | 12 (7.52) | 10.93 (5.96) | -0.35 | 0.73 | |
| **Δ Tremors OFF** | 1.72 (2.86) | 2.16 (2.22) | -0.66 | 0.51 | |
| **Δ Bradykinesia OFF** | 4.87 (3.73) | 4.60 (2.35) | -0.01 | 0.99 | |
| **Δ Rigidity OFF** | 1.65 (1.54) | 1.80 (1.97) | -0.39 | 0.69 | |
| **Δ Axial OFF** | 4.52 (3.04) | 3.87 (3.48) | -0.65 | 0.52 | |
| **Δ PIGD OFF** | 2.31 (3.24) | 1.67 (2.47) | -0.26 | 0.79 | |
| **Δ Motor complication** | 1.72 (2.52) | 2.47 (2.61) | -1.31 | 0.19 | |
| **Δ Hoehn and Yahr OFF** | 0.45 (0.51) | 0.50 (0.460) | -0.51 | 0.61 | |
| **Δ Schwab and England ADL OFF** | -11.38 (8.33) | -14.67 (7.43) | -1.40 | 0.16 | |
| **Δ IPAQ** | -804.50 (484.57) | -1002.63 (637.37) | -0.77 | 0.44 | |

AOO = age of onset, DOI= duration of illness, LEDD= Levodopa equivalent daily dose, LDL= Low density lipoprotein, HDL= High density lipoprotein, MDS-UPDRS= movement disorder society – unified Parkinson’s disease rating scale PIGD= postural instability and gait disorder,ADL = activities of daily living, TUG= time up and go test, 10 MWT= 10 meter walking test , BBS = berg balance scale , NFOG-Q = new freezing of gait questionnaire, IPAQ = international physical activity questionnaire, MMSE=mini mental state examination ,NMSS = non-motor symptoms scale ,BDI = beck depression inventory ,PDQ_39= Parkinson’s disease questionnaire-39.

* p value is significant if<0.05

**Table S7: Correlations between progression of motor subscores with baseline demographic, clinical , Lab and imaging characterstics:**

| **Mean difference between baseline and 1 year follow up** | **Δ MDS-UPDRS-III OFF** | | **Δ Tremors OFF** | | **Δ Bradykinesia OFF** | | **Δ Rigidity OFF** | |
| --- | --- | --- | --- | --- | --- | --- | --- | --- |
|  | *Pearson* | *Sig* | *Pearson* | *Sig* | *Pearson* | *Sig* | *Pearson* | *Sig* |
| **Age^ꭝ^** | 0.021 | 0.892 | -0.058 | 0.706 | -0.013 | 0.932 | -0.167 | 0.278 |
| **AOO^ꭝ^** | -0.03 | 0.849 | -0.129 | 0.403 | -0.087 | 0.572 | -0.104 | 0.502 |
| **DOI^ꭝ^** | 0.177 | 0.25 | 0.278 | 0.067 | 0.109 | 0.48 | -0.006 | 0.967 |
| **Baseline MDS-UPDRS-OFF-Total score** | 0.054 | 0.725 | 0.203 | 0.187 | -0.007 | 0.964 | 0.102 | 0.509 |
| **Baseline MDS-UPDRS-III** | 0.098 | 0.525 | 0.211 | 0.17 | 0.053 | 0.735 | 0.098 | 0.529 |
| **Baseline PIGD OFF ^ꭝ^** | 0.09 | 0.56 | 0.106 | 0.495 | -0.017 | 0.911 | 0.121 | 0.434 |
| **Baseline Hoehn and Yahr OFF^ꭝ^** | 0.252 | 0.099 | 0.17 | 0.27 | 0.16 | 0.301 | 0.202 | 0.189 |
| **Baseline TUG OFF^ꭝ^** | 0.202 | 0.188 | 0.131 | 0.396 | 0.101 | 0.515 | 0.125 | 0.419 |
| **Baseline NFOG-Q OFF^ꭝ^** | 0.098 | 0.526 | 0.09 | 0.563 | 0.008 | 0.958 | 0.006 | 0.97 |
| **Baseline MMSE** | **-0.173** | **0.262** | **-0.162** | **0.294** | **-0.11** | **0.478** | **-0.008** | **0.957** |
| **NMSS total score Baseline** | -0.245 | 0.109 | 0.091 | 0.556 | -0.289 | 0.057 | -0.17 | 0.269 |
| **BDI Baseline** | -0.149 | 0.335 | 0.025 | 0.871 | -0.106 | 0.495 | -0.16 | 0.299 |
| **PDQ-39 total score Baseline** | -0.008 | 0.959 | 0.105 | 0.498 | -0.024 | 0.877 | 0.146 | 0.343 |
| **IPAQ total score Baseline** | 0.075 | 0.629 | -0.116 | 0.452 | 0.102 | 0.509 | 0.012 | 0.941 |
| **Baseline LEDD** | -0.062 | 0.69 | 0.096 | 0.533 | -0.149 | 0.335 | 0.077 | 0.619 |
| **HbA1c%^ꭝ^** | **0.015** | **0.926** | **-0.172** | **0.283** | **0.078** | **0.63** | **0.053** | **0.74** |
| **Uric acid (mg/dL)** | 0.129 | 0.429 | -0.071 | 0.661 | 0.05 | 0.761 | 0.102 | 0.531 |
| **Cholesterol (mg/dL)** | 0.199 | 0.217 | 0.124 | 0.445 | 0.246 | 0.127 | 0.054 | 0.74 |
| **Triglyceride smg/dL)^ꭝ^** | -0.053 | 0.745 | -0.074 | 0.648 | 0.056 | 0.731 | -0.148 | 0.363 |
| **LDL (mg/dL)** | 0.188 | 0.246 | 0.006 | 0.97 | 0.219 | 0.174 | 0.127 | 0.435 |
| **HDL (mg/dL)^ꭝ^** | -0.168 | 0.314 | -0.074 | 0.659 | -0.174 | 0.296 | -0.118 | 0.479 |
| **Fazekas total^ꭝ^** | -0.132 | 0.4 | -0.1 | 0.524 | -0.007 | 0.963 | -0.245 | 0.113 |
| **Shelten_total^ꭝ^** | -0.023 | 0.884 | -0.181 | 0.239 | 0.089 | 0.567 | -0.085 | 0.584 |

AOO, age of onset, DOI, duration of illness; MDS-UPDRS= movement disorder society – unified Parkinson’s disease rating scale PIGD= postural instability and gait disorder,ADL = activities of daily living, IPAQ = international physical activity questionnaire, MMSE=mini mental state examination, NMSS = non-motor symptoms scale, BDI = beck depression inventory, PDQ_39= Parkinson’s disease questionnaire-39
